# A Case of Colonic Intussusception Caused by Cecal Lymphangioma and Requiring Emergency Surgery

**DOI:** 10.7759/cureus.63029

**Published:** 2024-06-24

**Authors:** Mitsutoshi Okuda, Takahiro Yoshioka, Ryosuke Tsunemitsu, Hiroaki Inoeu, Ryo Inada

**Affiliations:** 1 Department of Gastroenterological Surgery, Kochi Health Sciences Center, Kochi, JPN

**Keywords:** laparoscopic colorectal surgery, adult lymphangioma, laparoscopic emergency surgery, adult intestinal invagination, intra-abdominal lymphangioma

## Abstract

Lymphangiomas are benign tumors of dilated lymphatic vessels often found in the head, neck, and axilla of children. Lymphangiomas rarely occur in the abdomen, much less in the colon of adults. Colonic lymphangioma can cause symptoms and signs such as abdominal pain and abdominal distension and complications of gastrointestinal bleeding and intussusception. Intussusceptions are rarer in adults than in children. Most cases of intussusception in adults have a distinct cause, and a substantial number are related to malignant tumors. Herein, we report a rare case of ileocecal intussusception caused by cecal lymphangioma that required emergency surgery.

A 40-year-old woman presented with severe abdominal pain of a two-day duration. Her abdomen was tender and showed muscle rigidity in the right lower quadrant. Contrast-enhanced CT of the abdomen revealed ileocecal intussusception and a cystic mass, which was the leading point, with no evidence of bowel strangulation. The patient underwent emergency laparoscopic surgery because she had severe abdominal pain and showed peritoneal irritation signs. During surgery, the cecum was found to have invaginated into the ascending colon, almost to the hepatic flexure. Laparoscopic ileocecal resection with central vascular ligation was performed without intraoperative reduction of the intussusception because the cystic lesion could have a malignant component. Upon inspection, the lesion was a 60 mm × 50 mm submucosal mass located in the cecum. It was filled with clear serous fluid and thin walls. Pathology revealed the cystic mass to be a cecal lymphangioma with no evidence of malignancy. The patient was discharged seven days after emergency surgery with no complications.

Our case adds to evidence that cecal lymphangiomas can cause colonic intussusception in adults. Although rare, the risk of colonic intussusception must be considered in the management of colonic lymphangiomas.

## Introduction

Lymphangiomas are congenital malformations of lymphatic tissues that are believed to develop when the lymphatic system fails to converge with the main venous system during embryogenesis [1]. About half of these lymphangiomas are present at birth, and the majority will keep growing until the age of two years. Most lymphangiomas occur in the neck and axilla [2]. Lymphangiomas in the abdomen are rare, comprising <10% of all lymphangiomas [3]. Most cases of lymphangioma in the abdomen are located in the mesentery; however, they can also occur in the gastrointestinal tract, spleen, liver, kidneys, and pancreas [3]. Patients with colonic lymphangioma may be asymptomatic but can experience nausea, vomiting, abdominal pain, diarrhea, obstruction, gastrointestinal bleeding, and protein-losing enteropathy [4]. Intussusception is one of the rare complications of colonic lymphangioma with few reports in literature.

Intussusception is rare in adults and comprises about 5% of all cases of intussusception. Unlike children, where 90% of cases are idiopathic, most cases of intussusception in adults are caused by a distinct cause [5]. The most frequent causes are tumors, both benign and malignant. The standard treatment of choice for colonic intussusception is en-bloc resection without reduction because a substantial number of cases are associated with malignancy [6].

Herein, we report a rare case of ileocecal intussusception caused by cecal lymphangioma in a 40-year-old woman who required emergency surgery.

## Case presentation

A 40-year-old woman presented after experiencing intermittent abdominal pain over a two-day period. Her abdomen was tender, with muscle rigidity in the right lower quadrant. She had a medical history of hypertension and a surgical history of cesarean section. Her regular medications included amlodipine.

Regarding vital signs, body temperature was 36.9 °C, blood pressure 105/65 mmHg, and pulse 61 beats per minute. Laboratory investigations showed no evidence of inflammation, cell lysis, or increased tumor marker levels. Her CRP level was 0.05 mg/dl, and her white blood cell count was 7220/μl with no leftward shift. Regarding tumor markers, levels of CEA and CA19-9 were 1.9 ng/ml and 15.8 U/ml, respectively. Since the patient was a young woman with no concurrent respiratory disease, blood gas analysis was performed using venous blood, and the venous blood gas test showed no evidence of acidemia or lactemia. The venous blood gas test results were as follows: pH, 7.419; arterial carbon dioxide tension (PaCO2), 40.6 mmHg; partial pressure of arterial oxygen (PaO2), 54.3 mmHg; bicarbonate (HCO3), 26.3 mmol/L; acute bilirubin encephalopathy (ABE), 1.6 mmol/L; anion gap, 11.3 mmol/L; and lactate, 0.6 mmol/L.

**Figure 1:**
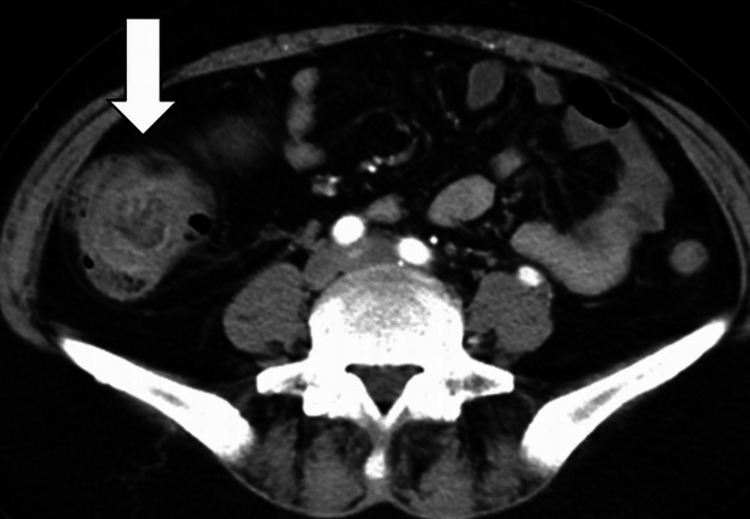
Contrast-enhanced CT of invagination of the ascending colon Contrast-enhanced CT revealed invagination of the ascending colon. No reduction in the contrast effect of the invaginated colon was observed.

An abdominal ultrasound showed a cystic mass in the ascending colon. Contrast-enhanced abdominal CT revealed the presence of an ileocecal intussusception and cystic mass (Figure 1).

**Figure 2:**
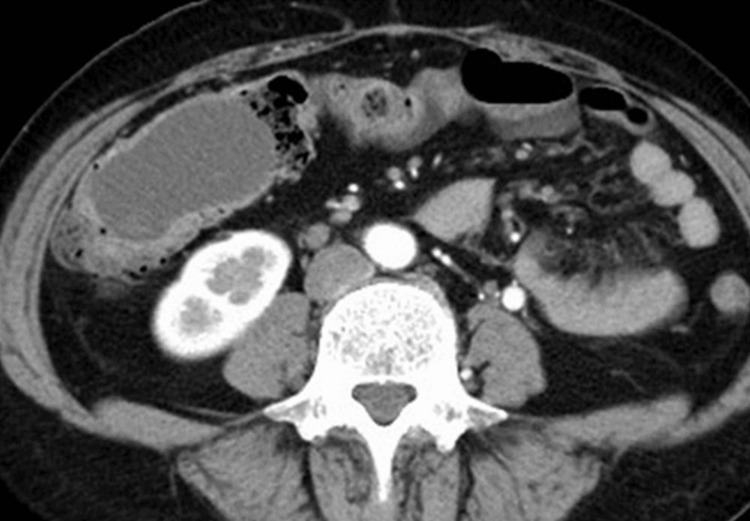
Contrast-enhanced CT image of a cystic mass The leading point of the colonic invagination was a cystic mass with a diameter of 50 mm. The mass was homogenous and had no solid components.

The cystic mass was approximately 50 mm in diameter and was the leading point of invagination, completely obstructing the lumen (Figure 2).

The contrast effect of the colon involved in the intussusception and the colon covering the cyst was not decreased, along with no evidence of pneumatosis, portal vein gas, and lymphadenopathy. Based on these CT findings, we concluded that it was unlikely that the patient was suffering from intestinal necrosis. The adoral colon was not distended, and mild fat-stranding signs were positive in the ileocecal region. However, no signs of ascites were present.

We made a preliminary diagnosis of colonic intussusception secondary to an ileocolic cystic mass. Urgent laparoscopic surgery was performed because the patient complained of severe abdominal pain. Additionally, peritoneal irritation signs picked up from the physical examination contributed to the need for urgent laparoscopic surgery. During surgery, the cecum had invaginated into the ascending colon, almost to the hepatic flexure. A decision was made to perform laparoscopic ileocecal resection with central vascular ligation. The ileocecal artery was ligated near the root because it was the feeding vessel for the tumor, and lymph node dissection was performed. Due to the possibility that the cystic mass could have a malignant component, we did not reduce the intussusception intraoperatively. Ileocecal region mobilization and central vascular ligation were performed intracorporeally. Thereafter, we made a small incision in the middle of the lower abdomen to extract and resect the ileocecal region. The dilation of the colon proximal to the intussusception was relatively small, and the patient was young with good nutrition status. Therefore, after the ileocecal resection, the remnant colon and terminal ileum were mechanically anastomosed extracorporeally without a temporary ileostomy.

**Figure 3:**
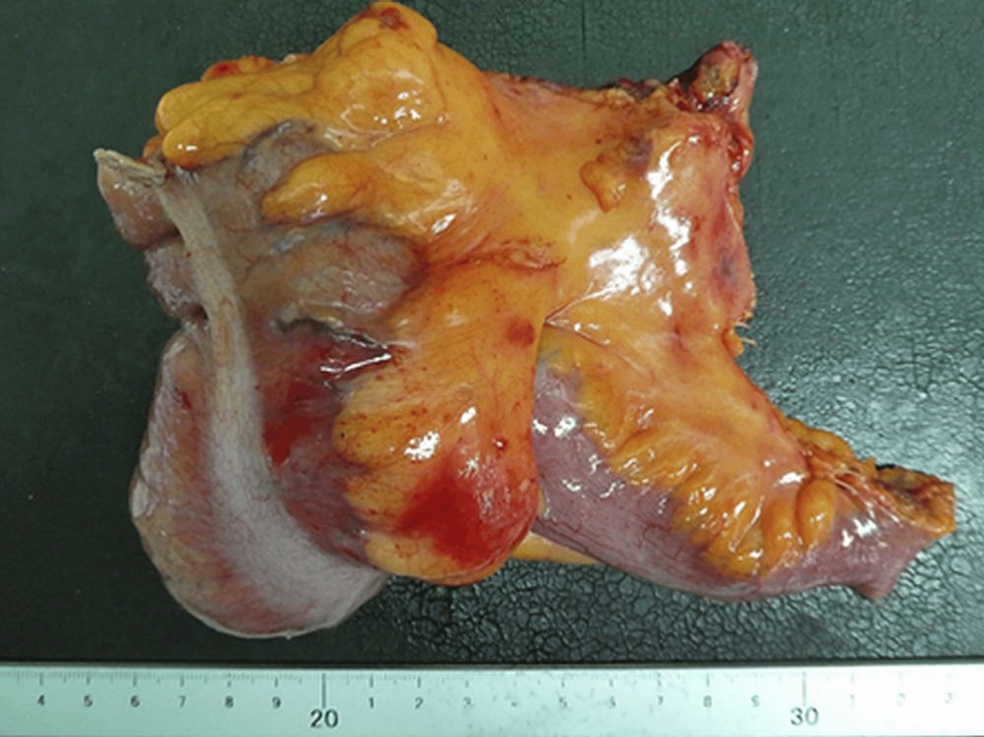
Macroscopic finding of the resected specimen A laparoscopic ileocecal resection with central vascular ligation was performed because of the possibility that the cystic mass may have a malignant component. The invagination was not reduced intraoperatively.

**Figure 4:**
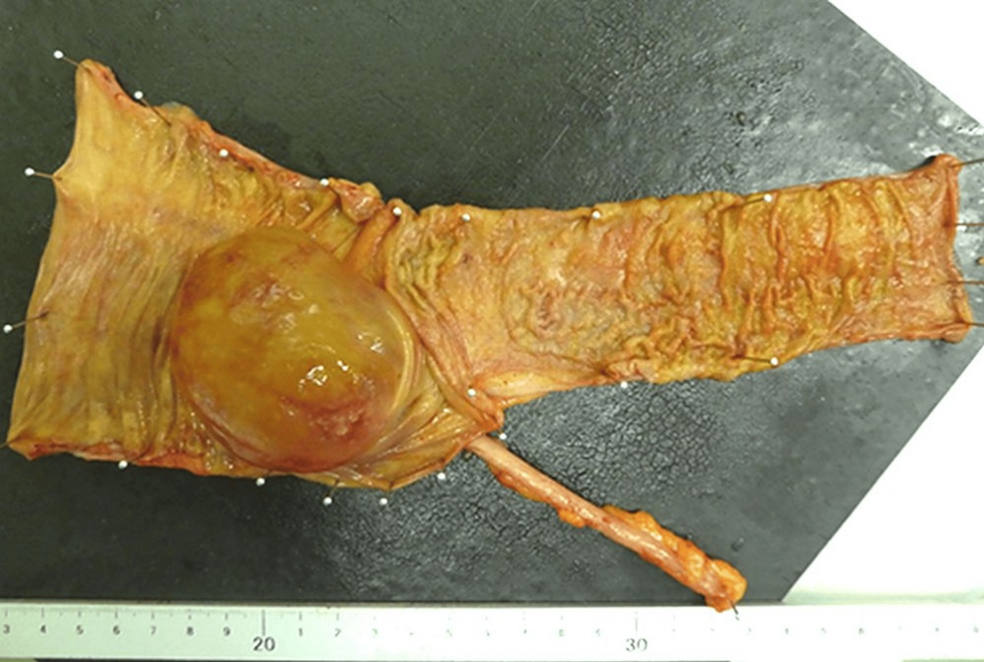
The resected ileocecal region (reduced) The specimen was opened to locate the lesion. The lesion was a 60 mm × 50 mm submucosal mass located in the cecum.

The resected specimen had a submucosal mass, measuring 60 mm × 50 mm, within the cecum filled with clear serous fluid and thin walls (Figures 3, 4).

**Figure 5:**
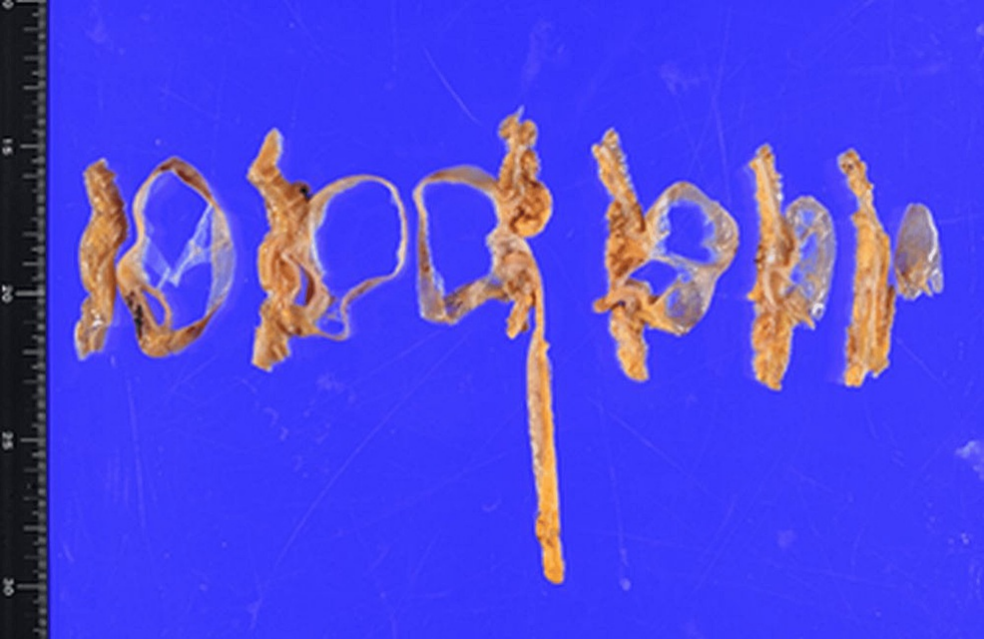
Gross pathological finding The cystic mass was filled with clear serous fluid and thin walls.

**Figure 6:**
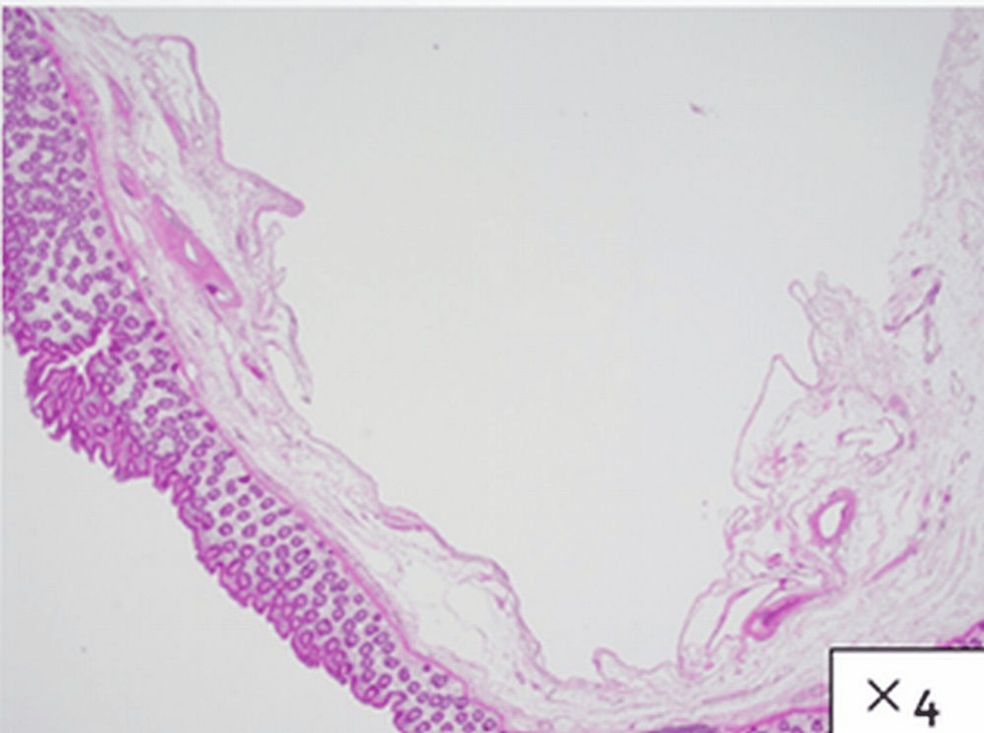
Pathological findings (HE) HE staining of the specimen revealed numerous dilated lymphatic vessels located in the submucosa covered by a single layer of endothelium. HE: hematoxylin and eosin

**Figure 7:**
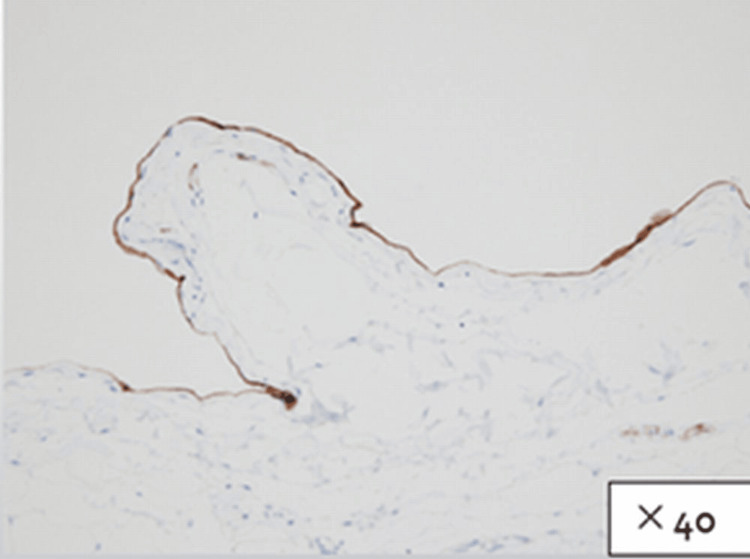
Pathological findings (D2-40) The single-layered endothelium covering the lesion stained positive for immunostaining D2-40.

Pathological studies showed the lesion to consist of numerous dilated lymphatic vessels located in the submucosa covered by a single layer of endothelium. The endothelium stained immunohistochemically positive for D2-40 and negative for CD34, CD31, AE1/AE3, calretinin, and CK5/6, confirming the diagnosis of a lymphangioma. No signs of malignancy were present (Figures 5-7).

The patient was discharged on postoperative day 7 without any complications.

## Discussion

Our case report adds to the limited number of reports of colonic intussusception caused by colonic lymphangioma in literature. To our knowledge, 10 cases of intussusception due to colonic lymphangioma have been reported in the English literature [7-16]. In six of these reports, the intussusception was caused by cecal lymphangiomas and comprised a majority of the lead point of intussusception. The other four cases included lymphangioma of the transverse colon, descending colon, ascending colon, and spontaneous multiple lymphangiomas [7,9,12,16].

In a review of 279 cases of colonic lymphangiomas in Japan, Matsuda et al. revealed that colonic lymphangioma most often originated in the transverse colon (n = 87), followed by the ascending colon (n = 72) [17]. Cecal lymphangiomas comprised only 13.5% (n = 34) of all cases. From a literature search of PubMed using the keywords “colon” and “lymphangioma,” we identified 76 papers, which included case reports and series. These papers reported 97 cases of lymphangiomas. Among the literature reporting on the positions of lymphangiomas, those reporting the cecum as a site formed 16.4% (n = 12) of the 73 cases of colonic lymphangiomas. Similar to findings by Matsuda et al., the cecum was found to be a rare site of origin for colonic lymphangiomas [17]. Although the number of cecal lymphangiomas is relatively small compared to other sites of origin among colonic lymphangiomas, lymphangiomas that caused intussusception mostly originated in the cecum. The tendency of lymphangiomas of the cecum, a site of origin that is relatively uncommon, to lead to intussusception remains unexplained. Possibly, since the cecum is anatomically a blind end, it easily becomes a leading point, as compared to other colonic positions [18].

According to the 10 reported cases of intussusception caused by colonic lymphangioma, the greatest diameter of the lymphangiomas ranged from 1.3 cm to 10 cm (median = 6.5 cm) [7-16]. The greatest diameter in our case was 6 cm, which was similar to the median diameter in the 10 cases. However, we identified a report of intussusception caused by a lymphangioma with the greatest diameter of 1.3 cm. Lymphangioma with a larger diameter was thought to have a higher risk of intussusception, but a lymphangioma with a larger diameter as small as about 1 cm has enough potential to cause intussusception.

No consensus on the management of colonic lymphangiomas has been reached, due to the relatively low incidence. In general, the management of colonic lymphangioma will depend on its location, size, growth speed, and symptoms [19]. By recommendation, asymptomatic cases and lymphangiomas with the greatest diameters (<2.5 cm) can be observed or endoscopically resected [19]. However, patients with lymphangioma may have an increased risk of colonic intussusception and should be informed of such risk. Additionally, colonic lymphangiomas have the potential to grow and invade adjacent structures, leading to severe complications [19]. Although colonic lymphangiomas are not known to undergo malignant transformation, the differential diagnoses of lymphangiomas may include malignancy [19,20]. As mentioned previously, lymphangiomas with a larger diameter as small as 1 cm have the potential to cause intussusception. For these reasons, surgical resection should be offered as one of the treatment options for asymptomatic patients. If the patient does not opt for surgery, the patient should at least be informed of the risk of potential complications.

Precise preoperative diagnosis of the cause of adult intussusception can be difficult. However, lymphangiomas would initially appear as submucosal cystic masses with differentials of lymphoma, schwannomas, lipoma, leiomyoma, carcinoid tumors, and GISTs [20]. Some of these differentials may have malignant components; therefore, en-bloc resection of the tumor and resection of surrounding lymph nodes without reduction of the invaginated lesion minimize the risk of intraluminal seeding of malignant cells and intravenous dissemination of tumor cells [6]. In the case of ileocecal lesions, the technical difficulty of ileocecal resection with complete vascular ligation is equivalent to that of simple ileocecal resection. Therefore, we believe intussusception caused by cecal lymphangioma would require en-bloc resection of the tumor and resection of surrounding lymph nodes.

## Conclusions

We report a rare case of colonic invagination caused by cecal lymphangioma and requiring emergency surgery. The risk of colonic invagination must be considered in the management of colonic lymphangiomas.
